# Acquisition and persistence of strain-specific methicillin-resistant *Staphylococcus aureus* and their determinants in community nursing homes

**DOI:** 10.1186/s12879-017-2837-3

**Published:** 2017-12-06

**Authors:** Nataliya G. Batina, Christopher J. Crnich, Dörte Döpfer

**Affiliations:** 1 Department of Industrial and Systems Engineering, University of Wisconsin-Madison, 3270 Mechanical Engineering Building, 1513 University Avenue, Madison, WI 53706 USA; 2 Department of Medicine, University of Wisconsin-Madison, 2500 Overlook Terrace, B5112E, Madison, WI 53705 USA; 3 William S. Middleton Veterans Affairs Hospital, 2500 Overlook Terrace, B5112E, Madison, WI 53705 USA; 4 Department of Medical Sciences, School of Veterinary Medicine, University of Wisconsin-Madison, 2027 Veterinary Medicine Building, 2015 Linden Dr, Madison, WI 53706 USA

**Keywords:** USA300 MRSA, Non-USA300 MRSA, Colonization, Risk factors, Acquisition, Persistence, Nursing home

## Abstract

**Background:**

Nursing home residents are frequently colonized with various strains of methicillin-resistant *Staphylococcus aureus* (MRSA) but the intra-facility dynamics of strain-specific MRSA remains poorly understood. We aimed at identifying and quantifying the associations between acquisition and carriage of MRSA strains and their potential risk factors in community nursing homes using mathematical modeling.

**Methods:**

The data was collected during a longitudinal MRSA surveillance study in six nursing homes in South Central Wisconsin. MRSA cultures were obtained from subjects every 3 months for up to one year. MRSA isolates were subsequently strain-typed by pulsed-field gel electrophoresis (PFGE), and their genetic similarity was established based on the Dice coefficients. Bayesian network analysis, logistic regression and elastic net were used to quantify the associations between acquisition and carriage of MRSA strains discriminated at 80% and 95% strain similarity thresholds and potentially modifiable resident characteristics including previous antibiotic exposure, comorbidity, medical devices, chronic wounds, functional and cognitive status and recent hospitalizations.

**Results:**

Absence of severe cognitive impairment as well as presence of a wound, device and severe comorbidity was associated with elevated probability of USA100 carriage although there was a variation based on the combination of those risk factors. Residents with severe comorbidity and cognitive status and presence of device and wound were identified as certain carriers of USA100 in our sample. Residents with a chronic wound were more likely to carry USA100 MRSA (OR = 2.77, 95% CI = 1.37–5.87). Functional status was identified as an important determinant of carriage of USA100 and USA300 strains. Comorbidity and cognitive status were the two factors associated with carriage of all clonal groups in the study (USA100, USA300 and USA1200).

**Conclusions:**

The combination of Bayesian network analysis, logistic regression and elastic net can be used to identify associations between acquisition and carriage of MRSA strains and their potential risk factors in the face of scarce data. The revealed associations may be used to generate hypothesis for further study of determinants of acquisition and carriage of selected MRSA subtypes and to better inform infection control efforts in community nursing homes.

**Electronic supplementary material:**

The online version of this article (10.1186/s12879-017-2837-3) contains supplementary material, which is available to authorized users.

## Background

Methicillin-resistant *Staphylococcus aureus* (MRSA) is a major cause of infection in health-care facilities and the community [1]. Infections due to MRSA are associated with higher morbidity, mortality and costs compared with infections caused by methicillin-susceptible strains [2–4]. Colonization with MRSA is known to increase the risk of a subsequent infection in hospital patients and nursing homes residents [5–7]. Nursing homes, where MRSA is endemic [8–11], may play an important role in the regional spread of MRSA [12–14]. The emergence of USA300 clone of MRSA in the community and its subsequent spread to healthcare settings in recent years [11, 15, 16] has become a serious problem due to its higher potential for virulence, persistence and transmissibility compared with healthcare-associated strains [17–19].

The dynamics of MRSA within nursing homes could be influenced by a number of factors. Studies, many of which were conducted in Veteran Affairs nursing homes or skilled-care facilities, demonstrated that poor functional status, comorbidity, invasive medical devices, chronic wounds, recent antibiotic exposure and hospitalization were risk factors for MRSA colonization in long-term care facilities [9, 10, 20–23]. However, the impact of these factors on the strain-specific dynamics of MRSA in community nursing homes remains poorly understood. Previously, we used Markov chain models to predict the steady-state distribution of residents colonized with USA300 and non-USA300 MRSA and to assess the impact of potential risk factors on strain-specific acquisition of MRSA in community nursing homes [24]. We found that antibiotic use in the previous 3 months significantly increased acquisition rates of strain-independent MRSA and non-USA300. The effect of antibiotic exposure on the acquisition rate of USA300 and the influence of other potentially modifiable resident characteristics on MRSA acquisition were quite pronounced, but the statistical significance at the 95% confidence level was not achieved. In addition, we employed compartmental and stochastic models in order to evaluate the epidemic potential of strain-specific MRSA and to assess the conditions for MRSA reduction and elimination from community nursing homes [25]. Our results suggested that, while MRSA elimination from nursing homes was unlikely in practice, considerable reductions in MRSA prevalence could be achieved through decolonization therapy that could sustain higher clearance rates over time. Based on our models, antibiotic use in the past 3 months elevated the prevalence of non-USA300 and USA300 MRSA in the facilities, but was unlikely to lead to an outbreak. Moreover, large-scale MRSA outbreaks were not predicted to occur in this setting. However, due to low numbers of observations in some subgroups, especially among subjects colonized with USA300 and exposed to risk factors, our capacity to ascertain whether the candidate risk factors for acquisition and durability of colonization differed by MRSA strain type was limited. Furthermore, while our compartmental and stochastic models assumed that acquisition of one strain was independent from carriage of the other strain, whether this assumption holds in reality is not known.

Bayesian networks have become an increasingly common method for modeling complex and uncertain data in many fields, including medicine [26–28]. A Bayesian network is a graphical representation of a joint probability distribution between entities of interest, the number of which can be too large to be modeled successfully using traditional approaches (e.g., regression analysis) [29, 30]. The elastic net (ENET), a new regularization and variable selection method, is another novel approach that gains popularity in modeling scarce data with many predictors [31]. In this study, we sought to use the combination of Bayesian networks, logistic regression and elastic net modeling to identify and quantify the associations between acquisition and carriage of MRSA strains and potentially modifiable resident characteristics in community nursing homes. The identified associations would aid in determining resident characteristics suggestive of a higher risk for acquisition or carriage of specific MRSA strains. The specific aims of this study were: (1) to identify subtypes of MRSA and candidate risk factors associated with acquisition and carriage of the pathogens using Bayesian network analysis; (2) to quantify the strength of the associations between acquisition and carriage of the MRSA subtypes and their potential risk factors using the combination of Bayesian network, elastic net and mixed effects logistic regression modeling.

## Methods

### Overview

A Bayesian network approach was used to examine the associations between candidate risk factors and acquisition and carriage of MRSA strains discriminated at the 80% and 95% strain similarity thresholds. Mixed effects logistic regression modeling and elastic net were subsequently applied to quantify the strength of the associations revealed by the network. All of the analyses were performed in R version 3.1.2 or higher [32]. The study was reviewed and approved by the Health Sciences Institutional Review Board of the University of Wisconsin-Madison.

### Data

The data were collected for a prospective longitudinal study of MRSA colonization in six community nursing homes (size, ≥ 60 beds) in south-central Wisconsin between February 2008 and October 2010 [33]. Four hundred and forty nine of the 851 residents approached (53%) provided written informed consent and participated in the study. Facility-level characteristics and MRSA trends in the study facilities have been previously described [24, 33]. The study subjects were screened for MRSA colonization at multiple anatomical locations at baseline and every three months for up to one year, provided the subjects remained in the facility. Surface cultures of the nares, skin of the axilla, groin and peri-rectal region were obtained using sterile Dacron-tipped swabs. Additional surface cultures were collected from open wounds and the insertion sites of non-urinary invasive devices, when applicable; urine specimens were obtained from subjects with indwelling urinary devices [24, 33]. MRSA specimens were enriched in trypticase soy broth supplemented with 6.5% NaCl and allowed to incubate for 24 h before plating onto Mannitol Salt agar (Remel, Lenexa, KS) containing cefoxitin (4 μg/mL) [33]. The methods used to construct the data set employed in this study have been previously described [24]. Subject exposures that might impact their colonization status were also obtained. These candidate risk factors were characterized as static, that is, ascertained at baseline only, and time-varying which were collected every 3 months. The static risk factors comprised comorbidity (*Comorb*), functional status (*Func*) and cognitive status (*Cogn*). For the purpose of this study, they were dichotomized into *non-severe* (coded as *0*) and *severe* (coded as *1*). The subjects with Charlson Comorbidity Index [34] score ≥ 3, Katz Activities of Daily Living (ADL) [35] score < 2 and Minimum Data Set (MDS) Cognitive Performance Scale (CPS) [36] score ≥ 5 were classified at a severe level of the corresponding factor, and at a non-severe level otherwise. Time-varying risk factors included antibiotic exposure within the previous 3 months (*AB*), hospitalizations in the previous 3 months (*Hosp*), presence of a chronic wound (*Wnd*) and presence of an invasive medical device such as indwelling urinary catheter, percutaneous feeding tube, central venous catheter, or tracheostomy (*Dev*). The time-varying risk factors were also used as dichotomous variables in our study (*Non-exposed* was coded as *0* and *Exposed* was coded as *1*). The data from the six study nursing homes were aggregated for the analysis, so that the findings of our study would be representative of the hypothetical “average” nursing home in Wisconsin.

For the purpose of this study, MRSA strains recovered from the study subjects were considered genetically distinct at the 80% strain similarity threshold if their pulsed-field gel electrophoresis (PFGE) banding patterns differed by 4–6 bands [37]. Seven unique strains at the 80% similarity threshold were identified in our study. For each of them, two dichotomous outcome variables were created. These variables indicated the occurrence of strain acquisition (transition from non-colonized to colonized) and carriage (continuous colonization) events. The variables were denoted by *T* (for acquisition) and *C* (for carriage) followed by 2 digits indicative of respective MRSA clonal groups in agreement with the CDC classification (i.e., “01” represented USA100 MRSA, “03” specified USA300, and “12” stood for USA1200 clonal group). For example, outcome variables *T01* and *C01* were created to denote acquisition and carriage of USA100, respectively. For each strain, an acquisition event was considered to occur at time *t > 0* if the strain was recovered at time *t* but not at time *t-1*. A strain-specific carriage event was assumed to occur at time *t = 0* if the strain was recovered during the baseline examination. For subsequent examinations, a carriage event for a strain was considered to occur at time *t* if the subject was colonized with the strain at time *t-1* and remained colonized with the same strain at time *t*. For each outcome variable, the event occurrence was coded as 1 and non-occurrence as 0. This data set was reduced by removing strain-specific acquisition and carriage variables for which the event occurred in less than 1% of the subjects (this corresponded to fewer than 4 event occurrences in the data set). The reduced data set retained acquisition variables for USA100 (*T01*) and USA300 (*T03*), and carriage variables for USA100 (*C01*), USA300 (*C03*) and USA1200 (*C12*). The counts of these events per facility and risk factor exposure are presented in Additional file 1: Table S1.

Notably, acquisition and carriage events of closely-related strains may get different designations when considered at different discriminatory thresholds. For example, if a subject carried strain X from USA100 clonal group at time *t*, but only strain Y from the same clonal group was recovered at time *t + 1*, this event at time *t + 1* would be classified as carriage at the 80% strain similarity threshold and as acquisition at a higher discriminatory threshold. To assess the impact of this phenomenon on the associations between strain-specific acquisition and carriage events and candidate risk factors, the analysis of the strains discriminated at the 95% similarity threshold was also performed. In this study, MRSA isolates were considered genetically distinct at the 95% strain similarity thresholds if their PFGE banding patterns differed by 1–3 bands [37]. A total of 75 unique strains at the 95% similarity threshold were identified in the study. As done with the isolates at the 80% strain similarity threshold, two dichotomous outcome variables were created for each unique MRSA isolate identified at the 95% threshold. The variables were coded as *T* (for acquisition) or *C* (for carriage) followed by a 4-digit sequence. The first two digits were indicative of the respective MRSA clonal group, and the other two digits were assigned based on the genetic similarity to isolates maintained in the University of Wisconsin Infectious Disease Research Laboratory [37]. For example, outcome variables *T0101* and *C0101* were created to denote acquisition and carriage of the strain from USA100 clonal group. Likewise, the data set was reduced by removing the outcome variables that occurred in less than 1% of the subjects. The reduced data set contained 11 strain-specific acquisition variables with 4–12 event occurrences and 16 carriage variables with 4–38 event occurrences (Additional file 2: Table S2).

### Associations at the 80% similarity threshold

**Fig. 1:**
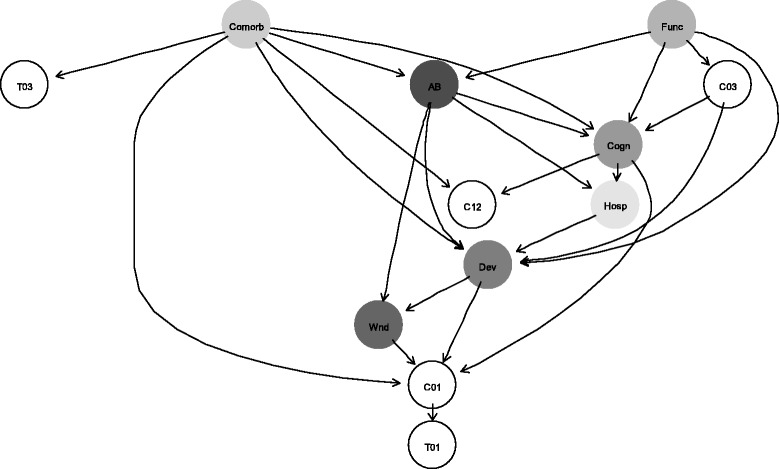
Averaged Bayesian network that includes all potential risk factors and events for MRSA clonal groups. The network was built by averaging 1000 networks learned from bootstrap resampling of the data. The averaged network included only significant arcs. *T* and *C* followed by a 2-digit sequence indicate acquisition and carriage events for the associated strain, respectively (e.g., *T01* and *C01* denote acquisition and carriage for USA100, *T03* and *C03* for USA300). *AB*, antibiotic use in the previous 3 months (0 = Non-exposed, 1 = Exposed); *Hosp*, hospitalizations in the previous 3 months (0 = Non-exposed, 0 = Exposed); *Dev*, invasive device (0 = Non-exposed, 1 = Exposed); *Wnd*, wound (0 = Non-exposed, 1 = Exposed); *Comorb*, comorbidities (0 = Non-severe, 1 = Severe); *Func*, functional status (0 = Non-severe, 1 = Severe); *Cogn*, cognitive status (0 = Non-severe, 1 = Severe)

To assess probabilistic dependencies between strain-specific acquisition and carriage events and potential risk factors, we used Bayesian network models. Bayesian networks are probabilistic graphical models that represent conditional dependencies between a set of random variables using directed acyclic graphs [29]. In Bayesian networks, random variables are represented by nodes (or vertices), and the conditional dependencies are shown by directed arcs that connect the nodes. For structure learning, we used model averaging to build a network containing only significant arcs [29]. The averaged network was constructed by applying bootstrap resampling to the data to learn a set of 1000 network structures. The arcs that appeared with higher frequency than the significance threshold estimated from the graphs were retained in the averaged network. The graph structure of each of the 1000 networks was learned with the score-based hill-climbing algorithm [29]. In this algorithm, the goodness of fit is assessed by the network score. The search begins with an empty network, and arcs are added, removed or reversed one at a time until the network score is no longer improving. Akaike information criterion (AIC) was used as a score function in the algorithm. The established network structure was used to estimate conditional probabilities of the strain-specific acquisition and carriage events. Candidate risk factors closely related to the strain-specific acquisition and carriage events were identified from the network with the so-called Markov blankets. The Markov blanket of a node in a Bayesian network comprises its parent nodes, children nodes and all other nodes that share a child with this node [29] (e.g., in Fig. 1, *Func* is the parent node of *C03*, *Dev* and *Cogn* are the children nodes of *C03*, and the nodes *AB*, *Comorb*, *Func*, and *Hosp* share a child with *C03*). The Markov blanket of each strain-specific acquisition and carriage event was determined from the averaged network and was used to quantify the strength of the associations between MRSA strains and potential risk factors. Focusing further analysis on the Markov blankets of the strain-specific events reduced the dimension of the model by removing non-informative factors [38]. The *bnlearn* package in R version 3.1.2 or higher [32] was used to fit Bayesian networks and to determine Markov blankets.

To quantify the strength of the association between strain-specific acquisition and carriage events and candidate risk factors, mixed effects logistic regression (MELR) models with logit link function were fitted. The outcome variables were acquisition and carriage events for MRSA clonal groups. Specifically, those were acquisition and carriage of USA100 (*T01* and *C01*, respectively), acquisition and carriage of USA300 (*T03* and *C03*, respectively), and carriage of USA1200 (*C12*). The independent variables in each original model were the Markov blankets of the corresponding outcome variable (the network is shown in Fig. 1). The models accounted for variability between the facilities by including the facilities variable, *Facility*, as a random effects term for the intercept. Different observations on the same resident were assumed independent in our study, where 128 subjects (29%) had only baseline observation [24]. The likelihood ratio test was used in the model building, and statistical significance was declared at 95% confidence level. Multilevel bootstrapping with 5000 replications was performed to derive 95% percentile confidence intervals (CI) for the estimated model coefficients. The coefficients of the significant predictors and their bootstrapped 95% CI’s were exponentiated to obtain odds ratios (OR) and their 95% CI’s. Exponentiated model intercepts denoted the odds of the outcome occurrence in the reference group (that is, when all predictors in the model were fixed at 0). The R (version 3.1.2 or higher) package *lmer* was used for model fitting [32].

In addition, the associations between strain-specific acquisition and carriage events at the 80% similarity and their candidate risk factors were quantified using the elastic net modeling approach. The elastic net is a regularization and variable selection method that produces sparse models with good prediction accuracy when the number of predictors is much higher than the number of observations [31]. In our ENET models, the strain-specific acquisition and carriage events served as outcome variables, while the covariates included the Markov blankets of the respective outcome variables and *Facility*. Cross-validation with 485-folds and α = 0.5 (the parameter controlling the penalty) was used to find the optimal tuning parameter λ. The number of folds in cross-validation was chosen to be about a third of the number of observations. The model coefficients were estimated based on the optimal tuning parameter at which the minimal mean-squared error (MSE) was achieved. The ENET models were fitted by using the *glmnet* package in R version 3.1.2 or higher [32].

### Associations at 95% similarity threshold

To obtain a preliminary understanding of the overall dependence structure between strain-specific transition and carriage events and candidate risk factors, we constructed a minimal BIC forest. The minimal BIC forest is a graphical model that represents the optimal forest for the data in which the penalized likelihood criterion, Bayesian Information Criterion (BIC), is optimized [39]. The nodes of the forest denote random variables. The edges between the nodes identify variables that are conditionally dependent given the other variables in the model. Likewise, the absence of edges between nodes identifies conditionally independent variables given the other variables. The minimal BIC forest was found and plotted by using the *gRapHD* package in R version 3.1.2 or higher [32]. Each vertex in the plot was determined by using the algorithm proposed by Fruchterman & Reingold [40] with 5000 iterations. To further our understanding of the associations between the potential risk factors and strain-specific acquisition and carriage events for MRSA strains at the 95% similarity threshold, we built an averaged Bayesian network, similarly to the network that involved clonal groups.

In order to explore the associations between each individual potential risk factor and strain-specific acquisition and carriage events while attenuating the impact of other risk factors, we considered 7 Bayesian networks, each of which included a single potential risk factor and all acquisition and carriage events. Similarly to constructing the Bayesian network that included all potential risk factors, each of the 7 networks was built by applying bootstrap resampling to data to learn a set of 1000 network structures and building an averaged network that included only significant arcs. In each averaged network, the Markov blanket of the potential risk factor was determined. To quantify the strength of the association between each potential risk factor and the strain-specific events that formed its Markov blanket, we attempted to employ logistic regression. However, logistic regression models could not be fitted successfully due to small number of observations and relatively large number of predictors. Elastic net models were fitted instead. In those models, the potential risk factor was considered a dependent variable, while the independent variables were strain-specific acquisition and carriage events that formed the Markov blanket of the risk factor, and *Facility*. Similarly, to derive the optimal tuning parameter λ, cross-validation with 485-folds and α = 0.5 was performed. The optimal tuning parameter at which the minimal mean-squared error (MSE) was achieved was employed to estimate model coefficients. The elastic net models were fit by using the *glmnet* package in R version 3.1.2 or higher [32].

## Results

### Associations at the 80% similarity threshold

**Fig. 2:**
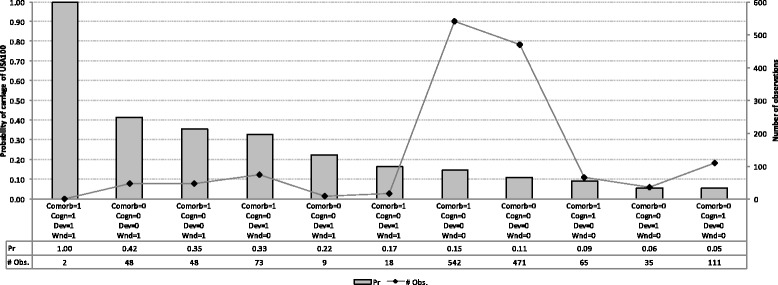
Conditional probabilities of carriage of USA100 derived from the Bayesian network depicted in Fig. 1. Conditions that correspond to a probability of 0 are not displayed. The secondary axis shows the total number of observations with respective combinations of risk factors. Pr, estimates of conditional probabilities; # Obs., the total number of observations with respective combinations of risk factors; *Comorb*, comorbidity (0 = Non-severe, 1 = Severe); *Cogn*, cognitive status (0 = Non-severe, 1 = Severe); *Dev*, presence of invasive device (0 = Non-exposed, 1 = Exposed); *Wnd*, presence of wound (0 = Non-exposed, 1 = Exposed)

Our Bayesian network revealed common patterns of conditional dependencies between acquisition and carriage events of MRSA clonal groups and their candidate risk factors (Fig. 1). The conditional probabilities of carriage of USA100, *C01*, estimated from the network are presented in Fig. 2 (the conditions associated with a zero probability are not shown). None of the conditional probabilities of occurrence of other events (*T01*, *T03*, *C03*, *C12*) exceeded 0.05 (data not shown). Thus, a resident with severe comorbidity and cognitive status who had an invasive device and chronic wound was certain to carry USA100 in our sample. Absence of severe cognitive impairment as well as presence of a wound, device and severe comorbidity influenced the probability of USA100 carriage although there was a variation based on the combination of those risk factors. For example, a resident with non-severe comorbidity and cognitive status who had a chronic wound but not a device was estimated to be a carrier of USA100 with probability of 0.42 in our sample.

This Bayesian network (Fig. 1) was also used to identify Markov blankets of the strain-specific acquisition and carriage events at the 80% strain similarity threshold. The Markov blankets for *T01* and *T03* consisted of a single factor*, C01* and *Comorb*, respectively. The Markov blanket for *C01* comprised *T01*, *Comorb, Cogn, Dev* and *Wnd*, while *AB*, *Comorb*, *Cogn, Dev*, *Func,* and *Hosp* formed the Markov blanket for *C03.* Lastly, the Markov blanket for *C12* included *Comorb* and *Cogn*.

Estimates for the significant predictors of acquisition and carriage events for MRSA clonal groups identified by MLER and ENET models from the respective Markov blankets are presented in Table 1. In this table, MLER estimates identify predictors that were statistically significant at the 95% confidence level. While logistic regression suggested statistically significant associations between the strain-specific acquisition and carriage events and their candidate risk factors, most of the 95% confidence intervals were extremely wide. One finding stood out: carriage of USA100 was more likely to occur in residents with a chronic wound than in those without it (OR = 2.77, 95% CI = 1.37–5.87).

**Table 1:** Coefficients derived from MELR and ENET models for strains discriminated at the 80% similarity threshold

Covariates	Elastic net (ENET)	Mixed effects logistic regression (MELR)			
	Estimate	Estimate (SE)^a^	Bootstrapped 95% CI^a^	OR (95% CI)^a^ (odds for Intercept)	*p*-value
*T01* (outcome)					
(Intercept)	−3.42	−3.45 (0.29)	(−4.46, −2.89)	0.03 (0.01, 0.06)	NA
*C01*	−5.83	NA	NA	NA	NA
*Facility*	0.09	0.58	(0.00, 1.37)	NA	NA
*C01* (outcome)					
(Intercept)	−2.53	−2.10 (0.30)	(−3.04, −1.41)	0.12 (0.05, 0.24)	NA
*T01*	−5.04	−20.30 (28.36)	(−82.16, −13.96)	0.00 (0.00, 0.00)	<0.001
*Cogn*	−0.88	−0.71 (0.30)	(−2.95, 0.72)	0.49 (0.05, 2.05)	0.010
*Comorb*	0.37	0.31 (0.16)	(−0.11, 1.04)	1.36 (0.89, 2.84)	NA^b^
*Dev*	0.26	NA	NA	NA	NA
*Wnd*	1.11	1.02 (0.21)	(0.31, 1.77)	2.77 (1.37, 5.87)	<0.001
*Facility*	0.15	0.66	(0.16, 0.92)	NA	NA
*T03* (outcome)					
(Intercept)	−4.83	−5.64 (0.76)	(−98.09, −4.25)	0.00 (0.00, 0.01)	NA
*Comorb*	0.46	1.11 (0.59)	(−0.99, 91.84)	3.03 (0.37, >1000)	0.047
*Facility*	NA	1.01	(0.00, 5.55)	NA	NA
*C03* (outcome)					
(Intercept)	−10.47	−5.14 (0.73)	(−29.75, −4.01)	0.01 (0.00, 0.02)	NA
*AB*	0.36	NA	NA	NA	NA
*Cogn*	1.15	1.34 (0.35)	(−24.83, 2.54)	3.81 (0.00, 12.70)	<0.001
*Comorb*	0.63	0.80 (0.34)	(−0.50, 23.66)	2.23 (0.61, >1000)	0.018
*Dev*	1.10	1.40 (0.36)	(−20.04, 2.34)	4.06 (0.00, 10.40)	<0.001
*Func*	5.92	NA	NA	NA	NA
*Hosp*	−0.43	NA	NA	NA	NA
*Facility*	0.14	1.48	(0.32, 3.23)	NA	NA
*C12* (outcome)^c^					
(Intercept)^c^	−7.40	−7.15 (1.35)/−34.55 (95.6)	(−783.01, −5.29)/(−715.76, −26.84)	0.00 (0.00, 0.01)/ 0.00 (0.00, 0.00)	NA
*Cogn*	2.33	2.41 (0.88)	(−447.79, 772.85)	11.16 (0.00, >1000)	0.005
*Comorb*	1.95	28.59 (95.60)	(21.23, 704.66)	>1000 (>1000, >1000)	0.003
*Facility* ^*c*^	−0.06	1.30/1.75	(0.00, 6.81) / (0.00, 7.39)	NA	NA

Covariates of each outcome variable (strain-specific acquisition or carriage events) represent Markov blankets of these variables. Covariates that were included in the Markov blankets of the outcome but not selected by the ENET or not statistically significant at the 95% confidence level in the MLER models are denoted by NA. The values in the OR and p-value columns that correspond to random effects terms of MELR models and the *p*-values for intercept terms are also denoted by NA’s. The *p*-values were obtained from the likelihood ratio test comparing two nested models, with and without the respective term

Logistic regression coefficients of the fixed effects terms (potential risk factor terms and acquisition and carriage of MRSA) are provided for level 1 (reference level is 0). Facility was used as a covariate in ENET models, and its coefficient is provided in the corresponding column. Standard deviations of random intercepts are provided in the columns that correspond to MELR

^a^MELR estimates and standard errors were derived from fitting the models to the data. Bootstrapped 95% CI’s are percentile CI’s obtained from multilevel bootstrapping with 5000 replications. OR’s are exponentiated model estimates, and respective 95% CI’s are exponentiated bootstrapped 95% CI’s

^b^Unable to estimate the p-value from the likelihood ratio test comparing two nested models because the model without the term does not converge

^c^The model with both *Cogn* and *Comorb* included did not converge. Two simpler models, each including one of the terms, were fitted. The first entry in the (Intercept) and *Facility* columns represent the values from the model with *Cogn*, and the second entry, separated by “/”, represent the values from the model with *Comorb*

*T01*, acquisition of non-USA300; *C01*, carriage of non-USA300; *T03*, acquisition of USA300; *C03*, carriage of USA300; *C12*, carriage of USA1200;

*AB*, antibiotic use in the past 3 months (0 = Non-exposed, 1 = Exposed); *Hosp*, hospitalizations in the past 3 months (0 = Non-exposed, 1 = Exposed); *Dev*, presence of invasive device (0 = Non-exposed, 1 = Exposed); *Wnd*, presence of wound (0 = Non-exposed, 1 = Exposed); *Comorb*, comorbidity (0 = Non-severe, 1 = Severe); *Func*, functional status (0 = Non-severe, 1 = Severe); *Cogn*, cognitive status (0 = Non-severe, 1 = Severe);

*OR* Odds ratio, *CI* Confidence interval, *SE* Standard error

In elastic net models, presence of chronic wound or invasive device and severe comorbidity were positively associated with carriage of USA100, while severe cognitive status appeared to be a protective factor. Notably, presence of wound showed a stronger association with carriage of USA100 compared to other factors. Among the factors positively associated with carriage of USA300, functional status appeared to be more pronounced. Carriage of USA300 was negatively associated with hospitalizations in the past 3 months. Acquisition of neither strain was associated with carriage of another strain.

### Associations at the 95% similarity threshold

**Fig. 3:**
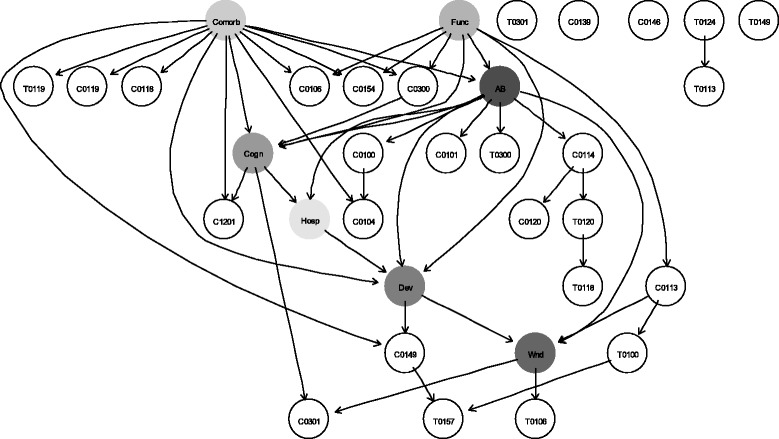
Averaged Bayesian network of potential risk factors and strain-specific acquisition and carriage events. The network was built by averaging 1000 networks learned from bootstrap resampling of the data. The averaged network included only significant arcs. *T* and *C* followed by a 4-digit sequence indicate acquisition and carriage events for the associated strains discriminated at the 95% similarity threshold, respectively. *AB*, antibiotic use in the previous 3 months (0 = Non-exposed, 1 = Exposed); *Hosp*, hospitalizations in the previous 3 months (0 = Non-exposed, 1 = Exposed); *Dev*, invasive device (0 = Non-exposed, 1 = Exposed); *Wnd*, wound (0 = Non-exposed, 1 = Exposed); *Comorb*, comorbidities (0 = Non-severe, 1 = Severe); *Func*, functional status (0 = Non-severe, 1 = Severe); *Cogn*, cognitive status (0 = Non-severe, 1 = Severe)

A preliminary dependence structure between strain-specific acquisition and carriage events and their candidate risk factors revealed by the minimal BIC forest is shown in Additional file 3: Figure S1. The graph suggests that the candidate risk factors play an important role in the spread and persistence of most MRSA strains discriminated at the 95% similarity threshold. A Bayesian network that shows conditional dependencies between strain-specific acquisition and carriage events of MRSA at the 95% similarity threshold and their potential risk factors is presented in Fig. 3. The Bayesian network and minimal BIC forest revealed similarities in the network structures. Moreover, the Bayesian networks for both 80% and 95% similarly thresholds appear qualitatively similar (Fig. 1 and Fig. 3, respectively).

An example of the Bayesian network that involved a single candidate risk factor (*AB*) and strains discriminated at the 95% similarity threshold is presented in Additional file 4: Figure S2. Elastic net analysis that was used to identify associations between strain-specific acquisition and carriage events and a single candidate risk factor resulted in the selection of covariates and their coefficients shown in Table 2. The total of 5 covariates was not selected by the ENET models from the Markov blankets of the candidate risk factors (*C0104* and *T0113* in relation to *AB*, *C0114* in relation to *Hosp*, *T0157* in relation to *Dev*, and *T0100* in relation to *Wnd*). *Hosp* was associated with acquisition and carriage events of strains from USA100 clonal group only, while other risk factors were associated with the events for strains from both USA100 and USA300 clonal groups. Carriage of the strain from USA1200 clonal group, *C1201*, was negatively associated with *AB* and positively associated with *Comorb*, *Func*, and *Cogn*.

**Table 2:** Coefficients of ENET models for strains discriminated at the 95% strain similarity threshold

Covariates (strain-specific events)	Estimated coefficients associated with the outcome variables (candidate risk factors)						
	*AB*	*Hosp*	*Dev*	*Wnd*	*Comorb*	*Func*	*Cogn*
(Intercept)	−0.56	−1.69	−2.25	−2.30	−0.39	0.75	−1.39
*C0100*	0.68	0.38	0.69	–	0.50	0.69	–
*T0100*	–	−1.65	–	NA	–	–	1.10
*C0101*	1.17	–	–	–	–	–	–
*C0104*	NA	–	–	–	−1.75	–	–
*C0106*	–	–	–	–	−0.68	1.12	–
*T0106*	0.99	1.45	–	3.34	–	–	–
*C0113*	–	–	0.06	1.43	–	3.47	−4.40
*T0113*	NA	–	–	1.25	–	–	–
*C0114*	0.96	NA	–	–	–	–	–
*C0118*	–	–	0.61	–	1.65	–	–
*C0119*	–	–	1.94	–	2.71	3.08	–
*T0119*	–	–	–	–	2.55	–	–
*C0120*	–	−1.56	–	–	–	–	–
*T0124*	–	–	–	0.17	–	–	–
*C0139*	–	–	–	–	1.09	–	–
*C0146*	–	–	–	1.49	–	−1.13	–
*C0149*	–	–	2.05	–	1.48	–	–
*C0154*	−0.54	–	–	–	2.91	−2.29	–
*T0157*	–	–	NA	–	–	–	–
*C0300*	0.55	–	1.19	0.52	0.78	3.70	0.97
*T0300*	0.54	–	–	–	–	–	–
*C0301*	–	–	1.20	1.95	–	3.15	1.53
*T0301*	–	–	–	–	–	2.99	–
*C1201*	−1.28	–	–	–	2.85	2.99	6.96
*Facility* ^a^	0.09	NA	NA	NA	0.13	0.03	−0.15

In these models, candidate risk factors served as dependent variables, while strain-specific acquisition and carriage events and *Facility* were included as independent variables. The rows represent the acquisition and carriage events which were a part of the Markov blanket of at least one potential risk factor. The columns display the estimates of the covariates selected by the models from the Markov blankets of the respective potential risk factors. NA’s denote the strain-specific events included in the Markov blanket of a risk factor but not selected by the model, while dashes signify the events that were not in the Markov blankets of the respective risk factors (e.g., *C0104* was in the Markov blanket of *AB*, but was not selected by the model; *T0100* was not in the Markov blanket of *AB*)

^a^Facility was included into the models as explanatory variable in addition to Markov blankets of the outcomes

*T* and *C* followed by a 4-digit sequence indicate acquisition and carriage events for the associated strain, respectively

*AB*, antibiotic use in the past 3 months (0 = Non-exposed, 1 = Exposed); *Hosp*, hospitalizations in the past 3 months (0 = Non-exposed, 1 = Exposed); *Dev*, presence of invasive device (0 = Non-exposed, 1 = Exposed); *Wnd*, presence of wound (0 = Non-exposed, 1 = Exposed); *Comorb*, comorbidity (0 = Non-severe, 1 = Severe); *Func*, functional status (0 = Non-severe, 1 = Severe); *Cogn*, cognitive status (0 = Non-severe, 1 = Severe)

## Discussion

Our study used the combination of Bayesian network analysis, logistic regression and elastic net modeling approaches to determine and quantify the associations between the acquisition and carriage of MRSA strains and their potential risk factors in community nursing homes in Wisconsin. The Bayesian networks that considered strains at the 80% and 95% similarity thresholds revealed a qualitatively similar structure of conditional dependencies between strain-specific acquisition and carriage events and potentially modifiable resident characteristics. To our knowledge, it is the first study that used predictive models to investigate the relation between acquisition and carriage of strain-specific MRSA and their determinants in community nursing homes.

Our results indicated that residents with a chronic wound were more likely to carry USA100 MRSA compared with residents free of wounds. This finding may suggest the presence of a chronic wound as a significant predictor of carriage of USA100 MRSA. The presence of wound was found to be a risk factor for MRSA carriage in other studies [41]. It may also be probable, however, that carriage of USA100 increases the risk for developing a chronic wound. More research is needed to study the direction of the association between carriage of USA100 MRSA and the presence of chronic wounds. Functional status appeared to be an important determinant for carriage of USA300 and USA100 strains in our study. This is in agreement with other studies conducted in nursing homes that identified functional status as a risk factor for MRSA colonization as well [22, 42]. The conditional dependencies revealed by the Bayesian networks highlight the associations between potentially modifiable resident characteristics and strain-specific acquisition and carriage of MRSA. Thus, the knowledge about resident cognitive status, comorbidity, presence of wound and device informs the likelihood of carriage of USA100 in our network. One of the important utilities of this approach can be informing targeted screening for MRSA in nursing homes (i.e., screening residents who are at a higher risk for carrying MRSA). Many hospitals are now pursuing targeted screening [43] in place of universal screening to reduce the costs. More research is needed to establish risk factors for MRSA colonization in nursing homes.

The limitations of our study stem largely from the scarcity of data and from modeling assumptions. The small number of observations available for strain-specific acquisition and carriage events, especially at the higher discriminatory threshold of 95%, coupled with scarcity of events for most risk factors, made some of the association estimates highly uncertain. To improve the predictive power of the models, the data from the six facilities were combined for the analysis. Our results therefore represent the hypothetical “average” nursing home. Combining the data did not allow us to describe the dynamics of MRSA within each facility. We did not account for variation between the residents within each facility due to the limited amount of observations at a resident level. Moreover, the proportion of the participants (53% of the approached residents) in the study nursing homes and subsequent attrition over time may limit the generalizability of the conclusions. Furthermore, the risk factor variables were dichotomized for the purpose of this study. Variable dichotomization was likely to reduce the discriminatory power of the models aimed at detecting the associations between the candidate risk factors and strain-specific events. For example, residents who had a single dose of antibiotics and those with routine antibiotic exposure over the previous 3 months were classified as exposed to antibiotics. Similarly, residents were classified as having severe or non-severe comorbidity, functional status and cognitive status based on the chosen cutoffs for the associated scores. That is, residents with a similar level of exposure to the risk factor of interest could be classified into different levels of this factor. However, we used the best available data to quantify the associations between MRSA strains and candidate risk factors with regards to their acquisition and persistence. More studies are needed to investigate such associations further. While we learned the network structure from the data, incorporating expert knowledge in the network may shed additional light into the determinants of acquisition and carriage of MRSA strains.

## Conclusions

We employed Bayesian networks, logistic regression and elastic net modeling approaches to study the associations between acquisition and persistence of MRSA strains and potentially modifiable resident characteristics in community nursing homes. The discovered associations may be used to generate hypotheses regarding risk factors for acquisition and persistence of MRSA strains in this setting for further research. Bigger data sets are needed to test these hypotheses in order to identify risk factors that contribute to acquisition and persistence of strain-specific MRSA in nursing homes.

## Additional files

Additional file 1: Table S1. Counts of event occurrences per facility and exposure to potential risk factors. (DOCX 35 kb)

Additional file 2: Table S2. Counts of acquisition and carriage for strains identified at the 95% strain similarity threshold. (DOCX 31 kb)

Additional file 3: Figure S1. Minimal BIC Forest for MRSA strains distinguished at 95% strain similarity threshold. The network shows the conditional dependence structure between strain-specific acquisition and carriage events and the candidate risk factors. (DOCX 15 kb)

Additional file 4: Figure S2. Averaged Bayesian network that includes recent antibiotic use and strain-specific acquisition and carriage events. The network was built by averaging 500 networks learned from bootstrap resampling of the data. The averaged network included only significant arcs. T and C followed by a 4-digit sequence indicate acquisition and carriage events for the associated strains discriminated at the 95% similarity threshold, respectively. AB, antibiotic use in the past 3 months (0 = Non-exposed, 1 = Exposed). (DOCX 17 kb)

## Abbreviations

CI: Confidence interval
ENET: Elastic net
MELR: Mixed effects logistic regression
MRSA: Methicillin-resistant *Staphylococcus aureus*
OR: Odds ratio
PFGE: Pulsed-field gel electrophoresis
