# Supplementary material for: Acquisition and persistence of strain-specific methicillin-resistant Staphylococcus aureus and their determinants in community nursing homes
Source: BMC Infect Dis. 2017 Dec 6;17:752. doi: 10.1186/s12879-017-2837-3 (PMC5719525; doi:10.1186/s12879-017-2837-3)
Supplement: Supplementary file 1 — Counts of event occurrences per facility and exposure to potential risk factors. (DOCX 35 kb) [file 12879_2017_2837_MOESM1_ESM.docx]

**Appendix Table A1.** Counts of event occurrences per facility and exposure to potential risk factors.

| **Event** | **Facility** | **Total counts** | **Counts among exposed to risk factors** | | | | | | |
| --- | --- | --- | --- | --- | --- | --- | --- | --- | --- |
|  |  |  | *AB* | *Hosp* | *Dev* | *Wnd* | *Comorb* | *Func* | *Cogn* |
| ***T01*** | 1 | 6 | 4 | 1 | 0 | 1 | 2 | 2 | 2 |
| ***T01*** | 2 | 13 | 7 | 0 | 1 | 0 | 7 | 12 | 2 |
| ***T01*** | 3 | 4 | 2 | 1 | 0 | 0 | 0 | 4 | 1 |
| ***T01*** | 4 | 18 | 9 | 1 | 1 | 2 | 10 | 15 | 2 |
| ***T01*** | 5 | 10 | 6 | 3 | 0 | 4 | 5 | 6 | 1 |
| ***T01*** | 6 | 1 | 1 | 1 | 0 | 0 | 1 | 1 | 0 |
| ***C01*** | 1 | 23 | 15 | 7 | 0 | 2 | 9 | 22 | 3 |
| ***C01*** | 2 | 41 | 24 | 8 | 8 | 15 | 23 | 25 | 0 |
| ***C01*** | 3 | 14 | 8 | 4 | 2 | 3 | 12 | 11 | 2 |
| ***C01*** | 4 | 81 | 39 | 5 | 16 | 17 | 50 | 66 | 1 |
| ***C01*** | 5 | 41 | 20 | 8 | 7 | 6 | 27 | 31 | 8 |
| ***C01*** | 6 | 12 | 4 | 2 | 0 | 1 | 10 | 9 | 0 |
| ***T03*** | 1 | 6 | 3 | 0 | 1 | 0 | 5 | 3 | 0 |
| ***T03*** | 2 | 0 | 0 | 0 | 0 | 0 | 0 | 0 | 0 |
| ***T03*** | 3 | 4 | 2 | 1 | 0 | 1 | 1 | 4 | 1 |
| ***T03*** | 4 | 1 | 1 | 0 | 1 | 0 | 1 | 1 | 0 |
| ***T03*** | 5 | 0 | 0 | 0 | 0 | 0 | 0 | 0 | 0 |
| ***T03*** | 6 | 4 | 3 | 2 | 2 | 0 | 4 | 3 | 0 |
| ***C03*** | 1 | 17 | 9 | 3 | 6 | 8 | 7 | 17 | 10 |
| ***C03*** | 2 | 1 | 1 | 0 | 1 | 0 | 0 | 1 | 1 |
| ***C03*** | 3 | 8 | 2 | 0 | 0 | 0 | 3 | 8 | 5 |
| ***C03*** | 4 | 2 | 0 | 0 | 0 | 0 | 2 | 2 | 0 |
| ***C03*** | 5 | 0 | 0 | 0 | 0 | 0 | 0 | 0 | 0 |
| ***C03*** | 6 | 19 | 15 | 2 | 7 | 2 | 19 | 19 | 0 |
| ***C12*** | 1 | 4 | 0 | 0 | 0 | 0 | 4 | 4 | 4 |
| ***C12*** | 2 | 0 | 0 | 0 | 0 | 0 | 0 | 0 | 0 |
| ***C12*** | 3 | 0 | 0 | 0 | 0 | 0 | 0 | 0 | 0 |
| ***C12*** | 4 | 0 | 0 | 0 | 0 | 0 | 0 | 0 | 0 |
| ***C12*** | 5 | 0 | 0 | 0 | 0 | 0 | 0 | 0 | 0 |
| ***C12*** | 6 | 2 | 1 | 0 | 0 | 0 | 2 | 2 | 0 |

*AB*, antibiotic use in the previous 3 months (0 = Non-exposed, 1 = Exposed); *Hosp*, hospitalizations in the previous 3 months (0 = Non-exposed, 1 = Exposed); *Dev*, invasive device (0 = Non-exposed, 1 = Exposed); *Wnd*, chronic wound (0 = Non-exposed, 1 = Exposed); *Comorb*, comorbidity (0 = Non-severe, 1 = Severe); *Func*, functional status (0 = Non-severe, 1 = Severe); *Cogn*, cognitive status (0 = Non-severe, 1 = Severe).
