# Supplementary material for: Acquisition and persistence of strain-specific methicillin-resistant Staphylococcus aureus and their determinants in community nursing homes
Source: BMC Infect Dis. 2017 Dec 6;17:752. doi: 10.1186/s12879-017-2837-3 (PMC5719525; doi:10.1186/s12879-017-2837-3)
Supplement: Supplementary file 2 — Counts of acquisition and carriage for strains identified at the 95% strain similarity threshold. (DOCX 31 kb) [file 12879_2017_2837_MOESM2_ESM.docx]

**Appendix Table A2.** Counts of acquisition and carriage for strains identified at the 95% strain similarity threshold.

| **MRSA strain** | **Acquisition, counts** | | | **Carriage, counts** | | |
| --- | --- | --- | --- | --- | --- | --- |
|  | Total events | Affected subjects | Affected facilities | Total events | Affected subjects | Affected facilities |
| 0100 | 11 | 11 | 5 | 29 | 13 | 5 |
| 0101 | - | - | - | 5 | 3 | 1 |
| 0104 | - | - | - | 7 | 4 | 2 |
| 0106 | 6 | 6 | 2 | 18 | 10 | 2 |
| 0113 | 9 | 9 | 2 | 20 | 12 | 1 |
| 0114 | - | - | - | 9 | 5 | 1 |
| 0118 | 8 | 8 | 2 | 9 | 5 | 2 |
| 0119 | 4 | 4 | 2 | 6 | 2 | 2 |
| 0120 | 7 | 6 | 1 | 9 | 6 | 1 |
| 0124 | 4 | 4 | 2 | - | - | - |
| 0139 | - | - | - | 6 | 5 | 4 |
| 0146 | - | - | - | 5 | 3 | 1 |
| 0149 | 6 | 6 | 2 | 16 | 8 | 3 |
| 0154 | - | - | - | 6 | 2 | 1 |
| 0157 | 4 | 4 | 1 | - | - | - |
| 0300 | 12 | 12 | 4 | 38 | 15 | 4 |
| 0301 | 4 | 3 | 1 | 6 | 3 | 1 |
| 1201 | - | - | - | 4 | 1 | 1 |

Strains with 3 or less event occurrences are not included.
