# Supplementary material for: Acquisition and persistence of strain-specific methicillin-resistant Staphylococcus aureus and their determinants in community nursing homes
Source: BMC Infect Dis. 2017 Dec 6;17:752. doi: 10.1186/s12879-017-2837-3 (PMC5719525; doi:10.1186/s12879-017-2837-3)
Supplement: Supplementary file 3 [file 12879_2017_2837_MOESM3_ESM.docx]

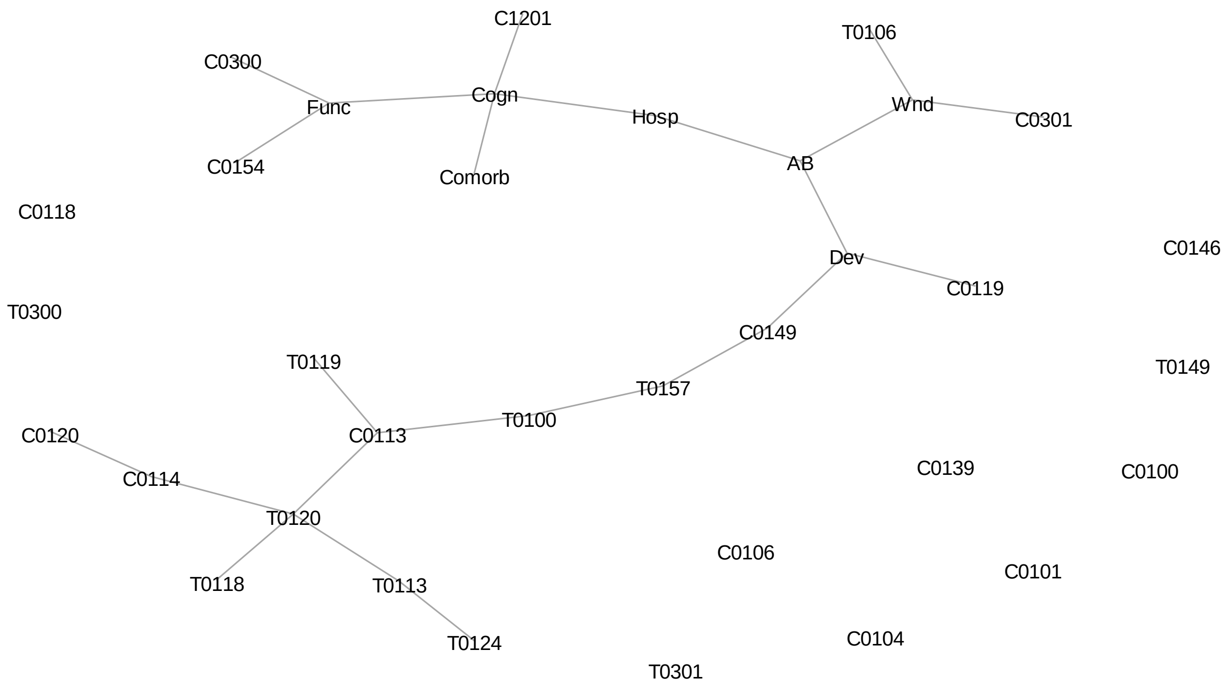
C1201
C0300
T0106
Func
Cogn
Hosp
Wnd
C0301
C0154
Comorb
AB
C0118
C0146
Dev
C0119
T0300
C0149
T0119
T0157
T0149
C0120
C0113
T0100
C0139
C0100
C0114
T0120
C0106
T0118
T0113
C0101
C0104
T0124
T0301
