# Supplementary material for: Acquisition and persistence of strain-specific methicillin-resistant Staphylococcus aureus and their determinants in community nursing homes
Source: BMC Infect Dis. 2017 Dec 6;17:752. doi: 10.1186/s12879-017-2837-3 (PMC5719525; doi:10.1186/s12879-017-2837-3)
Supplement: Supplementary file 4 [file 12879_2017_2837_MOESM4_ESM.docx]

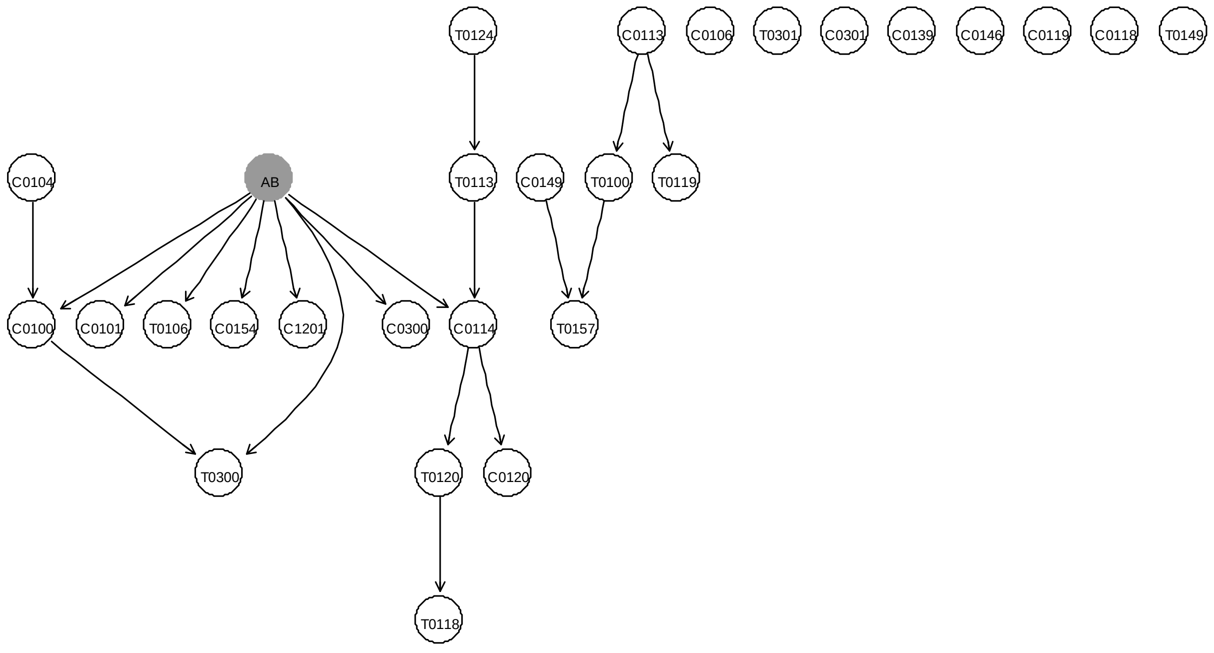
T0124
C0113
C0106
T0301
C0301
C0139
C0146
C0119
C0118
T0149
C0104
AB
T0113
C0149
T0100
T0119
C0100
C0101
T0106
C0154
C1201
C0300
C0114
T0157
T0300
T0120
C0120
T0118
